# The importance of recognizing worthlessness for suicide prevention in adolescents with Attention-deficit/hyperactivity disorder

**DOI:** 10.3389/fpsyt.2022.969164

**Published:** 2022-11-15

**Authors:** Luca Katzenmajer-Pump, Dániel Komáromy, Judit Balázs

**Affiliations:** ^1^Doctoral School of Psychology, Institute of Psychology, Eötvös Loránd University, Budapest, Hungary; ^2^Department of Developmental and Clinical Child Psychology, Institute of Psychology, Eötvös Loránd University, Budapest, Hungary; ^3^Department of Behavioral and Movement Sciences, Vrije Universiteit, Amsterdam, Netherlands; ^4^Faculty of Social and Behavioural Sciences, University of Amsterdam, Amsterdam, Netherlands; ^5^Department of Psychology, Oslo New University College, Oslo, Norway

**Keywords:** Attention-deficit/hyperactivity disorder, depression, general anxiety disorder, suicide, worthlessness

## Abstract

**Background:**

Attention-deficit/hyperactivity disorder (ADHD) is one of the most common psychiatric diagnoses among children and adolescents. Depression and general anxiety disorder (GAD) are often co-occurring with ADHD among children and adolescents. Previous studies have found that ADHD, depression and GAD are all strongly correlated with suicidal thoughts and planning.

**Aim:**

The current study aimed to further explore the association between ADHD, GAD and depressive symptoms as well as their association with suicidal thoughts and planning among adolescents.

**Method:**

Adolescents with ADHD diagnosis were involved from child psychiatry outpatient clinics and adolescents without a psychiatric treatment or diagnosis were enrolled from high schools in Hungary. The Mini International Neuropsychiatric Interview for Children and Adolescents was used to evaluate psychiatric symptoms and disorders as well as suicidal thoughts and planning. Regularized psychological networks were used to investigate the associations.

**Results:**

Altogether 185 adolescents (58 females and 127 males; mean age 14.79 years, SD = 1.48), 89 with ADHD and 96 without ADHD were enrolled. Depression symptom worthlessness was directly related to suicidal thoughts and planning, CI95 of the logit B between worthlessness and suicidal thought (0.72, 1.66). Both ADHD and anxiety were indirectly related to suicidal thoughts and planning through depression: CI95 of the logit B between being disorganized and feeling worthless is (0.38, 3.02), and CI95 of the logit B between being distressed and feeling worthless is (0.57, 2.52).

**Conclusions:**

This study draws the attention of clinicians to the importance of recognizing “worthlessness” for suicide prevention in adolescents with ADHD. Furthermore, the results support previous studies, whereby symptoms of depression and anxiety mediate the relationship between ADHD and suicidal thoughts and planning. These results highlight the importance of ADHD comorbidities with depression and GAD and their effect on suicidal thoughts and planning.

## Background

Attention-deficit/hyperactivity disorder (ADHD) is one of the most frequently diagnosed mental disorders among children and adolescents, with a prevalence of 4–6% (1, 2). It is associated with other mental disorders in 40–80% of cases, depending on the sample (3–5). In a treatment naive group of children and adolescents with ADHD, Keresztény et al. (5) found that the number of comorbid diagnoses with ADHD was significantly higher among adolescents than in children. Several researchers highlighted that comorbidity is one of the most important aspects of ADHD, and the comorbidities significantly affect the quality of life of these people (1, 6). Moreover, adolescents with ADHD and comorbidities have poorer long-term outcomes (7).

Recently, several researchers have focused on adolescents with ADHD and internalizing comorbid psychiatric problems (8, 9) and described a prevalence ranging from 12 to 50% for depression and 15 to 35% for anxiety (10). There can be several reasons behind this high comorbidity of ADHD and internalizing disorders (11–13). Evans et al. (12) described that ADHD predicted peer relationship problems, i.e., higher rates of victimization and also depression in the long term. According to Becker et al. (11), those adolescents with ADHD who were physically victimized had a greater association both with anxiety and depression. Mrug et al. (13) found that among adolescents with ADHD, peer rejection predicted long-term anxiety. Speyer et al. (14) aimed to investigate the developmental relations of internalizing and ADHD symptoms in a community sample of 1,387 children with a median age of 7 years at baseline and 9 and 11 years at the follow-ups with cross-sectional as well as longitudinal symptom networks. They found that ADHD shares reciprocal relations with internalizing symptoms and highlighted several potential bridge symptoms, i.e., cannot sit still, is restless and hyperactivity were found to be the strongest bridge symptoms to higher internalizing symptoms, whereas anxiety in children was the strongest antecedent for higher ADHD symptoms (14).

Based on previous research, suicidal thoughts and planning are strongly associated with ADHD (8, 9, 15–19). Giupponi et al. (17) point out that adolescents with ADHD indicated a higher risk of suicide than adolescents without ADHD. Biederman et al. (9) found that comorbid depression increased the risk of suicidal thoughts among adolescents with ADHD. Also, patients with diagnosed GAD and ADHD had higher risk of suicidality than ADHD patients without GAD (2). Levy et al. (18) found in their cross-sectional study that parent rated anxiety fully mediated the relationship between ADHD and suicidal thought and planning. Previous study of our research group found in a cross-sectional study based on multiple mediation analyses that the relationship between symptoms of ADHD and current suicidality was fully mediated by the symptoms of comorbid conditions: under age 12, significant mediators were symptoms of specific anxiety disorders, while in the adolescent age group symptoms of major depressive episode and dysthymia and substance abuse/dependence were found to be significant mediators (8). Mayes et al. (19) found that 78% of children and adolescents with ADHD, who had suicidal ideation experienced sadness and met the diagnosis of oppositional defiant disorder as well.

Brown et al. (20) found when they investigated first year undergraduate students: those who had ADHD showed a significantly higher level of both non-suicidal self-injury and suicidal ideation than those without ADHD. They also found that depression was a mediator between ADHD and suicidal thought and planning and non-suicidal self-injury (20). According to the previous research of our group, Balazs et al. (8) described that the symptoms of ADHD are associated with an increased risk for non-suicidal self-injury among adolescents, and affective and psychotic disorders and suicide mediated this relationship. Another study of our research group also found with a network approach that mood disorders, psychotic disorders and anxiety disorders mediated the path between attention/disruptive disorders and suicidality (21). Moreover, anxiety had the strongest association with ADHD (21). Furthermore, Taylor et al. (22) found that the ADHD symptom severity and suicidal thought and planning was significantly mediated by anxiety. Beard et al. (23) used network analysis for their causal system approach and found that when they looked at the individual symptoms of depression and anxiety, these were related more to other symptoms within the network, than between each disorder's network. They found that sad mood and worry were the two symptoms which were in the center of the network (23). Fried and Nesse (24) used a network approach as well to analyze depression symptoms and suggested analyzing each symptom separately within the system. According to Shim et al. (25), worthlessness and guilt had the strongest influence on suicidal thoughts and planning among depression symptoms in a network analysis. Rath et al. (26) used network approach as well to study suicide ideation and they found that it was related to depression and anxiety symptoms, as variables in the network. They also found that if participants scored high on anxiety, they scored high on depression as well (26).

In sum, our previous study, among others, revealed a triple-pathway model between attentive-disruptive disorders and suicidality: the association between the two variables were mediated by affective, psychotic, and anxiety disorders (5, 8, 9, 21). Our first aim was to confirm these findings on a different sample. Our second goal was to explore which facets of these variables are responsible for the mediation. Increased knowledge in this area can help to improve treatment focus and suicide prevention.

Based on the previous literature, we established the following hypotheses:

1. Both symptoms of anxiety and depression mediate the relationship between ADHD and suicidal thoughts and planning in adolescence.
2. There is a direct association between the symptoms of ADHD, anxiety and depression with suicidal thoughts and planning.

Furthermore, we aimed to identify which symptoms of depression and anxiety are the major contributors to suicidal thoughts and planning in adolescents with ADHD.

## Methods

### Procedure and participants

The entire protocol of the study and the characteristics of the sample have been previously published (27).

The Ethical Committee of the Medical Research Council, Hungary (ETT-TUKEB, project identification code: 50922-2/2017/EKU) approved the study. Participants and their parents/caregivers were informed about the study in written and oral forms. All participants and their parents/caregivers gave their oral consent, and participants older than 14 years and all parents/caregivers provided written informed consent.

An ADHD and a control group were enrolled. The inclusion criterion for both study groups was to be between the ages 13 and 18 years. A further inclusion criterion for the ADHD group was previous ADHD diagnoses by a clinician and a structured interview (see below). The control group's inclusion criteria were the absence of present or previous psychiatric diagnoses and treatment. The exclusion criterion for both groups was intellectual disability in the medical history. The participants for the ADHD group were from four different child psychiatric outpatient clinics in Hungary, while the control group consisted of three different high schools in Hungary. From the high schools around Baja, Hungary, 132 adolescent and their parents were contacted to participate in the research and at the end 96 adolescents and their parents completed the questionnaires (response rate: 72.73%).

### Measures

#### Demographic questionnaire

Demographic questions were asked of the participants' parents. These questions were developed explicitly for the current research. These included information about the parental education level, the economic activity of the parents, previous psychiatric diagnoses/treatment of family members, and the structure of the family.

#### The mini international neuropsychiatric interview for children and adolescents

The modified version of the Mini International Neuropsychiatric Interview for Children and Adolescents (M.I.N.I. Kid) has been used to assess twenty-four psychiatric diagnoses, including ADHD, major depressive episode and GAD (28, 29). The M.I.N.I. Kid is a structured diagnostic interview. The diagnostic criteria of the M.I.N.I. Kid are based on the Diagnostic and Statistical Manual of Mental Disorders fifth edition (DSM-5) (28). The original form of the M.I.N.I. Kid has a branching structure, which means, if the core symptoms of a disorder are not present, additional questions regarding the symptoms should not be asked. In our modified version of the M.I.N.I. Kid, we excluded the “branching logic” and in this way all the possible symptoms of a disorder were evaluated. The items in the M.I.N.I. Kid were binary, and we used the items for present symptoms only and we did not include questions regarding past disorders. The items of the disorder were placed into symptom groups rather than the disorder. Balázs et al. (29) validated the Hungarian version of the M.I.N.I. Kid and it had an adequate test-retest reliability, sensitivity and specificity. The interviewer posed the questions of the M.I.N.I. Kid to the adolescent. The M.I.N.I. Kid results were recorded by psychology and medical MSc and PhD students, who had completed a training course before the study, and during the study interviewers were regularly supervised to ensure inter-rater reliability.

### Statistics

Statistical analysis was performed using R (3.6.1 version, R Foundation for Statistical Computing, Vienna, Austria). As we had no prior assumptions about the exact paths through which different ADHD, anxiety and depression sub facets as well as suicidal ideation are interconnected, we did not use any models (such as mediation analysis or SEM) that predefine independent, mediator and dependent variables. Instead, we opted for regularized psychological networks that are widely used in psychiatry and clinical psychology (30–38).

Models in network psychometrics presume that observed variables have a causal influence on each other. In this perspective, correlations (edges) between variables (nodes) originates from mutual relationship between the symptoms rather than from the presence of latent variables (39). In this sense, partial correlation networks, since partial correlation is analogous to multiple regression coefficients, represent multicollinearity, on the one hand, and predictive mediation, on the other (39).

**Figure 1:**
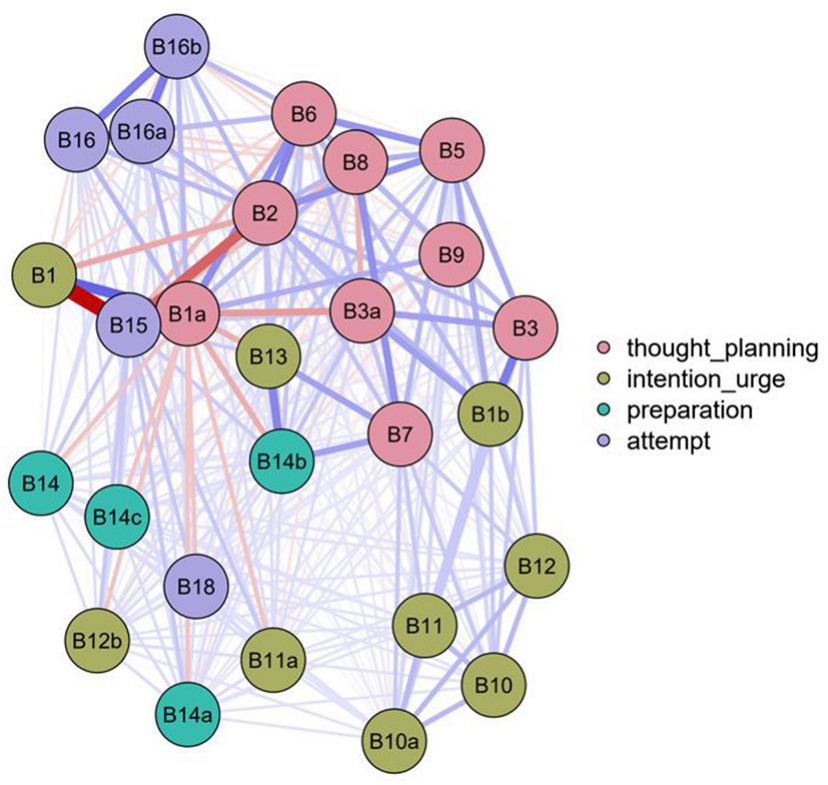
Unregularized Ising networks were estimated for all four facets of disorders separately to map the factor structure.

**Figure 2:**
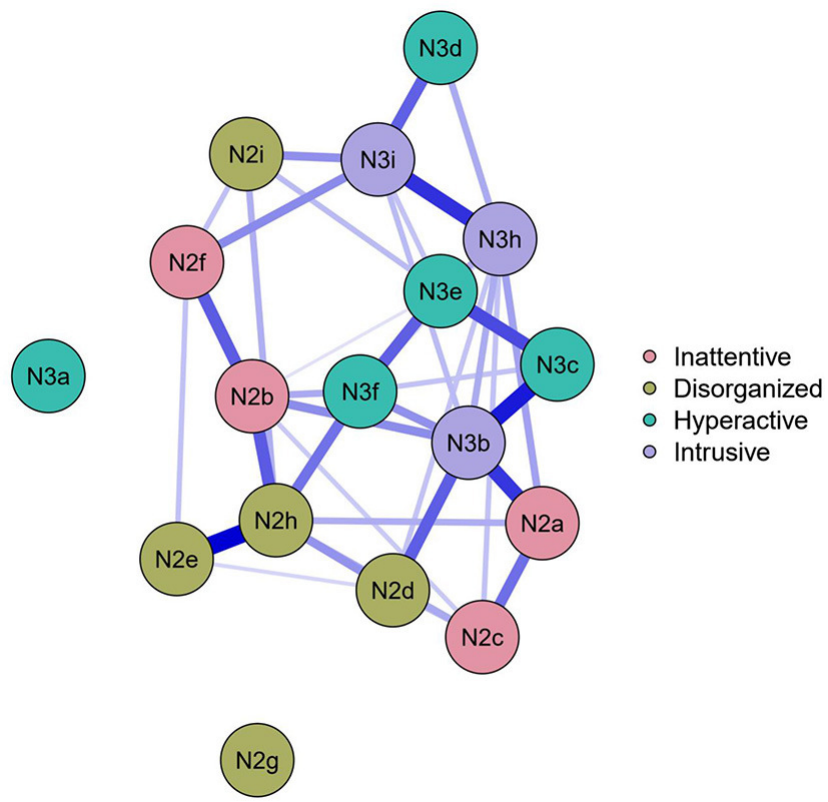
Regularized networks of the ADHD items colored by facets (before collapsing).

**Figure 3:**
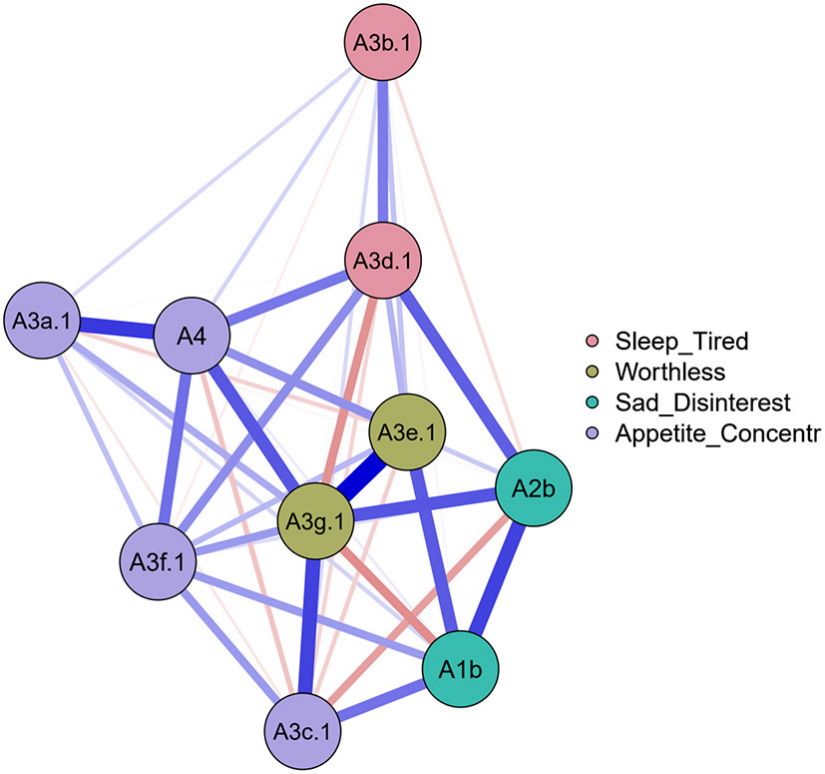
Regularized networks of the depression items colored by facets (before collapsing).

**Figure 4:**
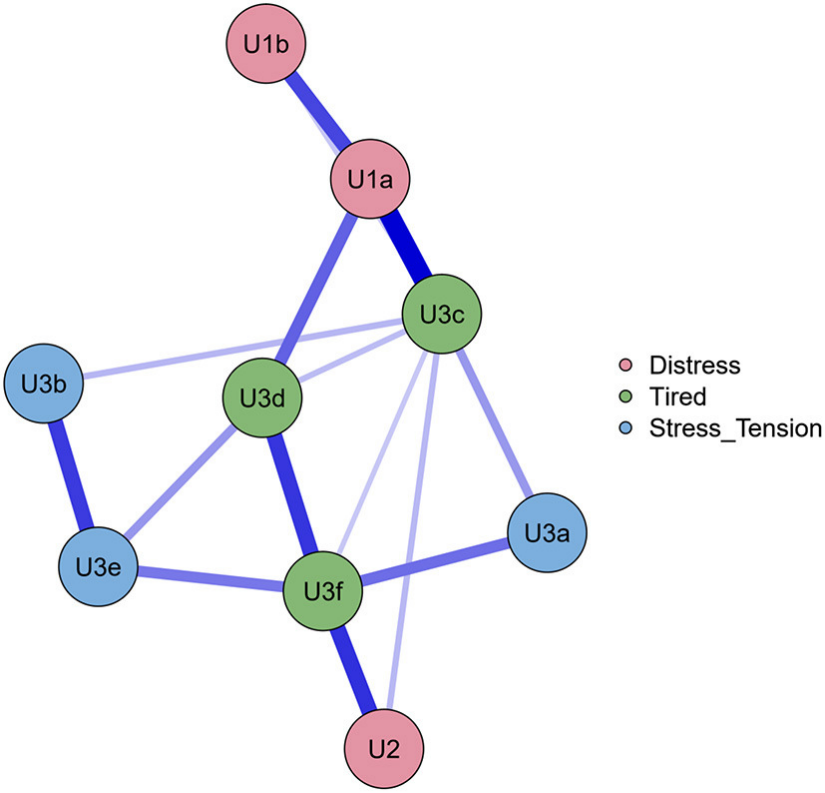
Regularized networks of the anxiety items colored by facets (before collapsing).

The alphanumeric nodes labels in the figures through the article represent questions regarding the symptoms of ADHD, anxiety, depression and suicide behavior as they are listed in the M.I.N.I Kid. Table 1 summarizes which items of the M.I.N.I Kid are represented by the alphanumeric node labels in Figures 1–4.

**Table 1:** M.I.N.I Kid items and the corresponding alphanumeric node labels in Figures 1-4.

**M.I.N.I. Kid topics**	**Alphanumeric node labels**	**M.I.N.I. Kid items**
Suicide thought and planning	B1a	Planned to hurt him or herself
	B2	Thoughts of being dead
	B3	Thoughts of hurt him or herself and dying as a result
	B3a	The number of times he or she thought of hurting him or herself with a possibility of dying
	B5	Had a method of killing her or himself
	B6	Knows what to use to kill her or himself
	B7	Knows where to kill her or himself
	B8	Knows when to kill her or himself
	B9	Knows what to finish before killing him or herself
Suicide intention and urge	B1	Had any accident before
	B1b	Wanted to die from an accident
	B10	Had the urge to finish killing her or himself
	B10a	Thoughts of finishing the plan of killing her or himself
	B11	Expected to die after hurting her or himself
	B11a	Wanted to die by suicide
	B12	Felt the need to kill her or himself
	B12b	Wanted to kill her or himself
	B13	Had difficulties to stop killing her or himself
Suicide preparation	B14	Prepared to kill her or himself but someone disrupted
	B14a	Prepared to kill her or himself but did not start
	B14b	Prepared to kill her or himself but then stopped before doing it
	B14c	Prepared to kill her or himself but someone stopped it
Suicide attempt	B15	Hurt her or himself without the wish of dying
	B16	Tried to kill her or himself
	B16a	Started to kill him or herself but then stopped and did not finish
	B16b	Start to kill him or herself but someone stopped it
	B16c	Tried everything to kill him or herself
	B18	Tried to die
ADHD groups Inattentive	N2a	Having problems with attention
	N2b	Having problems with focusing
	N2c	Being told not listening
	N2f	Avoiding programs which requires attention
ADHD groups Disorganized	N2d	Having problems following the exercise
	N2e	Having problems with organization
	N2h	Often being distracted easily
	N2i	Forgetting everyday tasks
ADHD groups Hyperactive	N3a	Moving a lot in the seat?
	N3b	Leaving the seat during class?
	N3c	Running around and climb on things?
	N3e	Being always “on the go”?
	N3f	Talking too much?
ADHD groups Impulsive	N3d	Feeling hard to play quite
	N3h	Feeling hard to wait
	N3i	Often interrupting people
Depression groups- Sleepy and tired	A3b1	Having problems to sleep
	A3d1	Feeling tired almost all the time
Depression groups Feeling of worthlessness	A3e1	Not feeling good about her or himself?
	A3g1	Feeling so bad to wish to die?
Depression groups Sad and disinterested	A1b	Feeling depressed or sad lately?
	A2b	Being bored and not motivated in anything lately?
Depression groups Appetite and loss of concentration	A3a1	Experiencing a change in appetite?
	A3c1	Talking slower than usually?
	A3f1	Having problems focusing?
	A4	Having problems from feeling depressed?
Anxiety groups distress	U1a	Being worried and anxious a lot in the past half year?
	U2	Worrying almost always lately?
Anxiety groups tired	U3c	Feeling tired lately?
	U3d	Having problems concentrating?
	U3f	Having problems sleeping?
Anxiety groups stress and tension	U3a	Not being able to sit still?
	U3b	Feeling tens lately?
	U3e	Feeling annoyed lately?

In the case of binary variables, Ising models (40) that are estimated through nodewise logistic regressions are used (41) meaning that iteratively, one variable is regressed on all others. In other words, an edge between two nodes depicts a significant relationship between two variables over and beyond the effect of all other variables. Accordingly, a lacking edge should suggest that two variables are conditionally independent.

Nevertheless, network edges are never zero due to sampling variation, but they can mirror spurious correlations or, in other words, false positives (42). As multiple testing (e.g., Bonferroni correction) results in a loss of power (42), to filter out spurious correlations, we used a regularization technique called “least absolute shrinkage and selection operator” [LASSO; (43)]. This technique maximizes the sum of the absolute value of the coefficients compared to an unregularized network. In a regularized network, all regression coefficients become smaller, and the small ones become zero, rendering the network sparser. Regularization happens through the lambda coefficient (41, 44): too low values may retain false positive edges, while too high values may remove true positives. Therefore, the goal is to maximize the number of true positives while minimizing the number of false positive ones (45, 46). The algorithm optimizes the number of edges by estimating various networks with different lambdas (39), and based on some criteria, it selects the best one. The Expected Bayesian Information Criterion [EBIC, (40)] has been shown to perform especially well in retrieving the true network structure (41, 45, 46). It is important to stress that the primary aim of LASSO is to minimize the number of false positives and reduce the number of false negatives only afterwards. Consequently, a lacking edge is not indisputable evidence for the lack of the relationship itself (47). It is analogous to the widely known issue in null hypothesis testing: not rejecting a null hypothesis does not necessarily mean that the null hypothesis holds. To implement the Ising model estimates both with and without EBIC LASSO regularization, the bootnet package (39) was used.

As a first step, we aimed to map the facets of each mental disorder. To do so, the first author manually categorized the items. Subsequently, unregularized Ising networks were estimated for each disorder that depicts the intercorrelation of the items. Instead of the DSM-5 based two ADHD symptom domains (i.e., hyperactivity-impulsivity and inattention), in the current analyses the ADHD part of the M.I.N.I. Kid was categorized into four specific symptom domains to best understand the relationship of the variables. Based on previous research, intrusive (48–51), and disorganization domains (22, 52–55) were added to the classical two domains as they were found to be significantly higher in patients with ADHD than the control group. Moreover, intrusive thoughts were found to be not only part of the ADHD symptomatology, but were also associated with suicidal thought and planning (51). Due to the low prevalence of suicidal thoughts and planning, we decided to use dichotomous variables representing these factors rather than summing the items. In this way, a factor takes the value of one if any of the pertaining items are one and takes the value zero otherwise. Out of the four factors of suicidal thought and planning (thought and planning, urge and intention, preparation, attempt) three had a prevalence lower than five percent. We analyzed only suicidal thought and planning.

As a second step, these binary factor variables were used in one large regularized network to map the conditional relationship between the different facets of ADHD, anxiety, depression and suicidal thought and planning. To obtain confidence intervals for the regression coefficients, bootstrapping was used with 1,000 bootstrap samples. Bootstrap results measure how often a given edge was estimated to be non-zero. Due to the regularization, all estimates are biased toward zero; hence, the entire sampling distribution is biased toward zero. It implies that the bootstrapped confidence intervals are not centered on the unbiased parameter value. Therefore, just as previously, if the bootstrapped interval contains zero, it could be that the unbiased one does not. However, if the bootstrapped CI does not overlap with zero, then the unbiased one does not overlap either (39).

After bootstrapping the results, the stability of our network was assessed. To do so, we estimated six network models with different hyperparameters and compared them along three commonly used centrality measures. In a model estimated with an EBIC LASSO, the gamma hyperparameter determines the sparsity of the network: higher values lead to the removal of more weak (potentially true positive) edges, while lower values may retain more (potentially false positive) correlations. We compared networks along strength, betweenness and closeness. Node strength reflects the sum of absolute edge weight associated with a certain node, betweenness counts how many times a given node is in the shortest path between two other nodes, and closeness calculates the inverse of the sum of distances from a node to all other nodes measuring how close a given node is to all other nodes (56). All of them are local measures, meaning that they characterize a node rather than the entire network. Consequently, the invariance of these measures, especially betweenness, would be indicative of (the robustness of) the mediator role of a given variable.

## Results

### Sample

In the present study, the total number of participants with ADHD were 89, and the number of participants in the controls were 96. Regarding gender, 58 females and 127 males participated in the study. The mean age was 14.79, SD = 1.48 and for gender: χ_2(1)_ = 37.87, *p* < 0.0001, Wilcoxon for age differences was: *W* = 3,885, *p* = 0.28. The proportion of participants with any suicidal thought was 92.97%.

We found a significant difference between gender between the two groups, in the ADHD group 92% were males and in the control group 54% were males [X${}_{\left(1\right)}^{2}$ = 39.32; *p* < 0.0001, V = 0.46].

### ADHD, depression, GAD and suicidal thoughts and planning

First, four variables were created by summing the items and Spearman correlations were calculated (see Table 2). The number of ADHD symptoms were significantly correlated with those of depression (ρ = 0.3, *p* < 0.001) and of suicidal thoughts and planning (ρ = 0.17, *p* = 0.02), but not with anxiety (ρ = 0.12, *p* = 0.11). Suicidal thoughts and planning were significantly related to depression (ρ = 0.3, *p* < 0.001), but not to anxiety (ρ = 0.06, *p* = 0.42). Anxiety was significantly associated with only depression (ρ = 0.34, *p* < 0.001).

**Table 2:** ADHD, GAD, depression, suicidal behavior and their correlations.

	**ADHD**	**Depression**	**Suicidality**
Depression	0.3 (*p* < 0.001)		
Suicidality	0.17 (*p* = 0.019)	0.3 (*p* < 0.001)	
Anxiety	0.12 (*p* = 0.111)	0.34 (*p* < 0.001)	0.06 (*p* = 0.422)

Second, unregularized Ising networks were estimated for all four disorders separately to map the factor structure. The network models are shown in Figures 1, 2, where the color of the nodes represents the categories determined by the authors. However, due to the very low prevalence of some items, the suicidal thoughts and planning network has not been proven to be reliable.

After the categorization, items were collapsed based on the facets; distributions are shown in Figures 3, 4. However, due to the low prevalence in the sample (6.49% for thought and planning, 3.78% for intention and urge, 1.08% for preparation and 1.62% for attempt), we kept only the thought and planning facet among the suicidal thoughts and planning factors.

**Figure 5:**
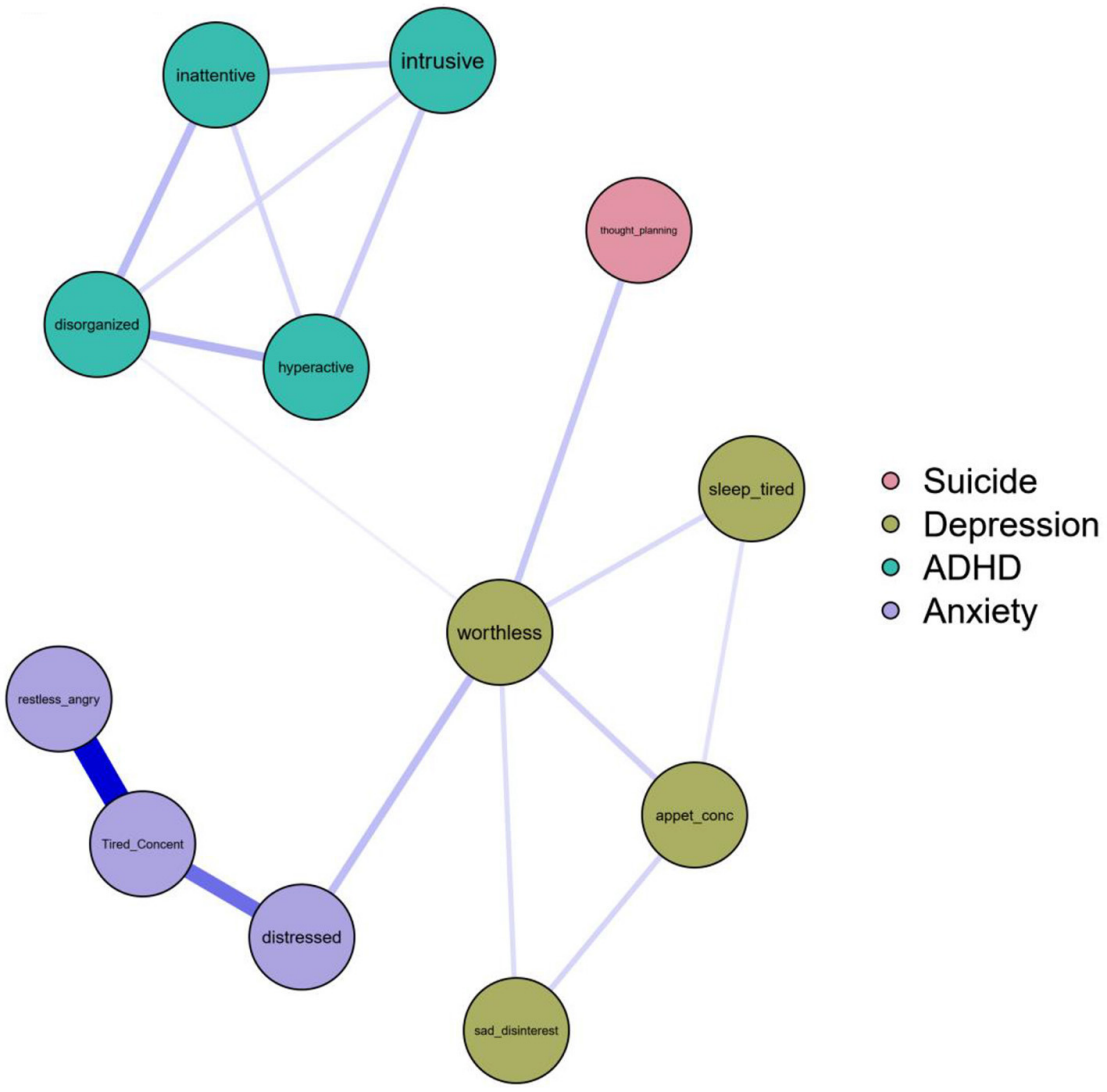
After creating the binary categories, the regulated Ising network model was estimated with one suicidality (thought and planning), three anxiety (distress, tiredness and concentration problems, restless and angry), four depression (sad and disinterested, appetite and concentration problems, sleepy and tired, worthless), and four ADHD (intrusive, inattentive, disorganized, hyperactive) variables.

Third, after creating the binary categories, the regulated Ising network model was estimated with one suicidality (thought and planning), three anxiety (distress, tiredness and problems with concentration, restlessness and anger), four depression (sad and disinterested, appetite and concentration problems, sleepy and tired, worthlessness), and four ADHD (intrusive, inattentive, disorganized, hyperactive) variables (Figure 5).

**Figure 6:**
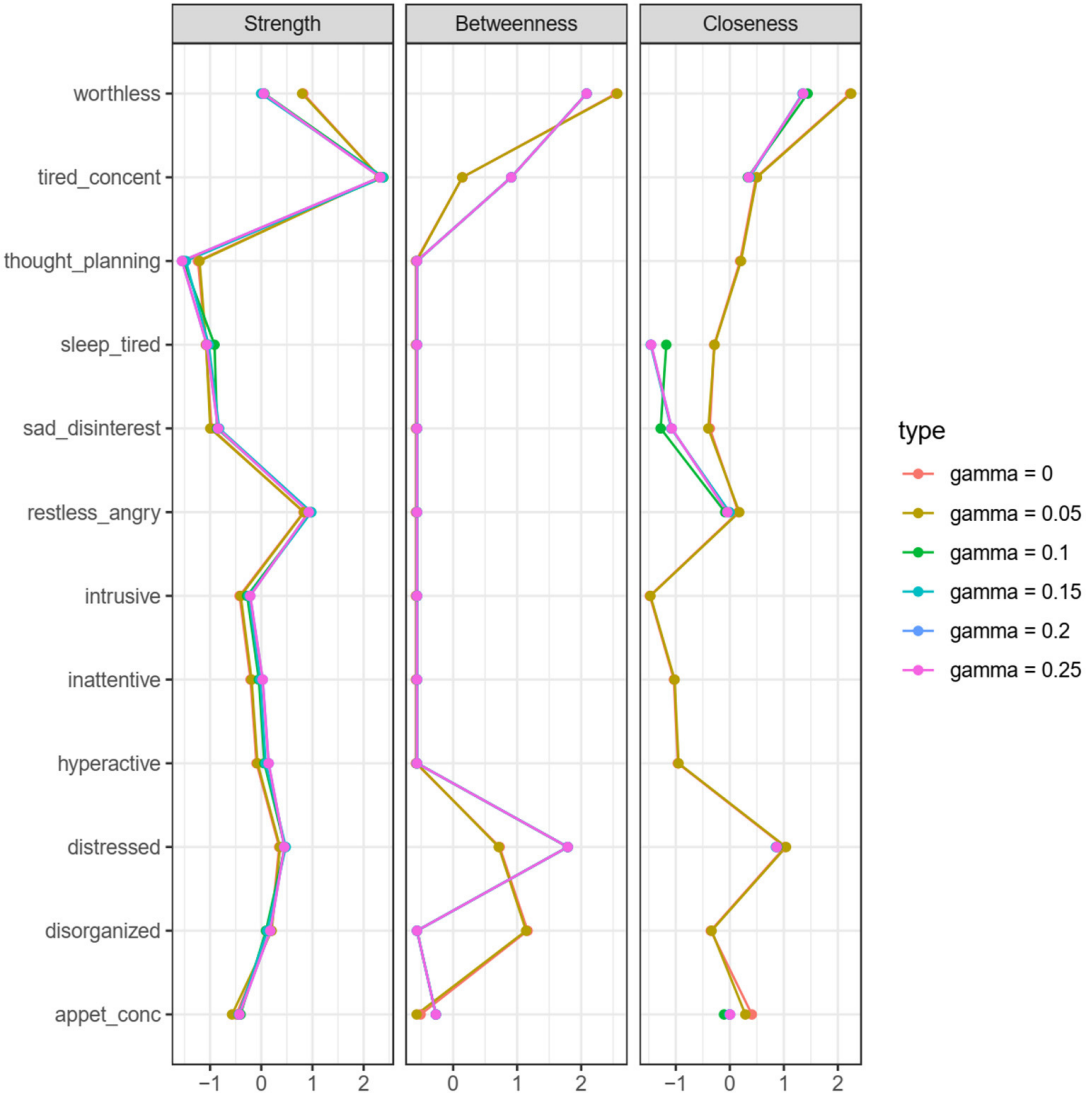
Robustness check—Comparisons of networks with different hyperparameters by three centrality measures.

Figure 6 assesses the robustness of our findings. The charts indicate that increasing the hyperparameter over 0.15 (removing the weak correlations) changes the betweenness estimate of the first mediating edge (between disorganization and worthlessness). Similarly, the closeness estimate of suicidal thoughts becomes infinite meaning that this variable is not connected anymore to the others. These results suggest that the mediating effect is relatively robust, however the effect size of the correlation between ADHD and depression is not large. The second path (between worthlessness and suicidal thoughts) seems to be robust as the betweenness estimates do not change considerably. The node strength estimates along the models are substantially unchanged; the plot suggests that only the removal of the weak edge between ADHD and depression modifies the absolute edge weight.

Although the zero-order correlations with suicidality were significant both for ADHD and depression, based on the network model, only depression was directly related to suicidal thoughts and planning, CI95 of the logit B between worthlessness and suicidal thought (0.72, 1.66). Both ADHD and anxiety were indirectly related to suicidal thought through depression: CI95 of the logit B between being disorganized and feeling worthless is (0.38, 3.02) and CI95 of the logit B between being distressed and feeling worthless is (0.57, 2.52). Hence, although hypothesis 1 is fully supported, hypothesis 2 is only partially supported.

## Discussion

To our knowledge, our current study is the first one which investigated the relationship of anxiety, depression, ADHD and suicidal ideation using a causal system perspective, where we identified the symptoms of the disorders and analyzed them using a network approach, following the analysis of Fried and Nesse (24). Furthermore, following the conception of suicidal behavior as a continuum (57), our study focused on suicidal thoughts and planning, as a possible early prevention phase of suicidal behavior.

In line with several previous findings, our first hypothesis was supported (8, 18, 24), whereas the symptoms of anxiety and depression mediate the relationship between ADHD and suicidal thoughts and planning. When anxiety and depression were controlled, the relationship between ADHD and suicidal thoughts and planning cease, meaning that only indirect association between ADHD and suicidal thoughts and planning was found in the current study. Cho et al. (58) also found that the comorbid conditions fully mediated the relation between ADHD and suicidality. Our research group previously found that the mediating symptoms between ADHD and suicidality in children and adolescents differ (8): symptoms of depression are essential mediators among adolescents, while symptoms of anxiety are essential mediators in children (8, 59, 60).

In the current study, we identified worthlessness and disorganized path of depression as the primary contributor to suicidal thoughts and planning. Worthlessness was first recognized in the Diagnostic and Statistical Manual of Mental Disorders 3rd Edition as one of the leading symptoms of depressive disorder (61). The frequency of worthlessness among current major depressed patients was reported as 70–80% in the USA (61). Furthermore, worthlessness as a symptom of depressive disorder was already found to be strongly related to suicidality in youth (62–65). However, the current study is the first one that highlights the role of worthlessness as a possible risk factor of suicidal thought and planning among adolescents with ADHD.

Our second hypothesis was only partially supported because we did not find a direct association between ADHD, anxiety and depressive symptoms with suicidal thoughts and planning. However, our results indicated that anxiety is associated with suicidal thoughts and planning but only through the worthlessness symptom of depression. There are few studies which focus on depressive symptoms as the mediator between anxiety and suicidal thoughts and planning among adolescents. Hill et al. (66) had similar findings, whereby they found that through depressive symptoms anxiety impacted later suicidal thoughts and planning. Thomas et al.'s (67) study indicated that among males, the influence of anxiety on suicidal thoughts and planning was mediated by depression.

The current study should be considered with the limitation of being cross-sectional, that is why we are not able to draw a causational relationship between the variables. Another limitation is that we found a low endorsement of suicidal thought and planning in our sample. Also, in the study male subjects dominated in the ADHD group. Finally, a potential selection bias could be present by looking at individuals with ADHD diagnosis in treatment.

In conclusion, we would like to highlight that our research supported previous findings since we also found that adolescents with ADHD had an association with suicidal thoughts and planning through depression and anxiety (8, 18). In line with previous studies, it is an important finding, because suicide is one of the leading causes of death among adolescents. The current study draws the attention of clinicians to the importance of screening suicidality among their patients with ADHD, with special focus on those who have comorbid depression and/or anxiety. The vital contribution of our research to previous findings is that the current study is among only a few which have explored all the symptoms of both depression and anxiety, which are the major risk factors for suicidal thoughts and planning. We would like to highlight that worthlessness was found to be among all depressive symptoms and was the only significant mediator between ADHD and suicidal thoughts and planning. Based on this result, we suggest that routine questions on the presence of the symptom of worthlessness should be inserted at the baseline and follow-up examinations of patients with ADHD. This could lead to suicide prevention as well. In the future it would be important to plan longitudinal research with gender balanced, treatment naïve sample.

## Data availability statement

The raw data supporting the conclusions of this article will be made available by the authors, without undue reservation.

## Ethics statement

The studies involving human participants were reviewed and approved by the Ethical Committee of the Medical Research Council, Hungary (ETT-TUKEB, project identification code: 50922-2/2017/EKU). Written informed consent to participate in this study was provided by the participants' legal guardian/next of kin.

## Author contributions

Conceptualization and basic aspects of the methodology used was accomplished by JB. The formal statistical analysis and visualization was performed by DK. Investigation, data curation, and project administration was made by LK-P. Resources were provided by JB. LK-P made the original draft preparation, while the review and editing was made by all authors. Supervision and funding acquisition was organized by JB. All authors provided final approval of the version of the manuscript for publication and agreed to be accountable for all aspects of the work.

## Conflict of interest

The authors declare that the research was conducted in the absence of any commercial or financial relationships that could be construed as a potential conflict of interest.

## Publisher's note

All claims expressed in this article are solely those of the authors and do not necessarily represent those of their affiliated organizations, or those of the publisher, the editors and the reviewers. Any product that may be evaluated in this article, or claim that may be made by its manufacturer, is not guaranteed or endorsed by the publisher.
